# Interpretable machine learning analysis for relationships between *Helicobacter pylori* infection and peripheral atherosclerosis: a retrospective cohort study

**DOI:** 10.3389/fcimb.2026.1693262

**Published:** 2026-02-11

**Authors:** Chenlu Li, Qingshi Lin, Liaoliao Yang

**Affiliations:** 1 Department of Gastroenterology, Affiliated Yueqing Hospital, Wenzhou Medical University, Wenzhou, China; 2 Interventional therapy Department, Affiliated Yueqing Hospital, Wenzhou Medical University, Wenzhou, China

**Keywords:** *Helicobacter pylori*, machine learning, mediation analysis, peripheral atherosclerosis, SHapley additive exPlanations analysis

## Abstract

**Background:**

Helicobacter pylori (*H.pylori*) has been implicated in peripheral atherosclerosis (PA); however, its predictive value for PA risk in large population-based cohorts remains insufficiently characterized.

**Objectives:**

This study aimed to evaluate assess the predictive contribution of *H.pylori* infection to PA risk, in combination with traditional clinical factors, using interpretable machine learning (ML) models.

**Methods:**

A retrospective cohort of 5,862 individuals undergoing routine health check-ups was analyzed. Demographic data, laboratory indices, and lower-extremity vascular ultrasound findings were collected to determine PA status and *H.pylori* infection. Key risk factors were identified through univariate and multivariate logistic regressions. Subgroup and restricted cubic spline (RCS) analyses were applied to evaluate effect modification and nonlinear associations. Fourteen ML algorithms were developed and evaluated using the area-under-curve (AUC), sensitivity, accuracy, specificity, positive-predictive-value (PPV), negative-predictive-value (NPV), F1-score, Youden’s index, and calibration. Models were subsequently retrained using regression-identified variables to assess stability. SHapley Additive exPlanations (SHAP) analysis was employed to interpret feature importance from across models. Longitudinal and mediation analyses were conducted to explore temporal relationships and the potential mediating role of the triglyceride-glucose (TyG) index.

**Results:**

*H.pylori* infection was independently associated with PA (odds ratio (OR)=5.27, 95%CI=4.27-6.54, *P*<0.001), alongside male (OR = 5.88, 95%CI=4.22-8.29, *P*<0.001), smoking (OR = 2.11, 95%CI=1.67-2.67, *P*<0.001), and elevated low-density lipoprotein levels (OR = 2.10, 95%CI=1.47-3.04, *P*<0.001). Subgroup and RCS analyses demonstrated consistent and nonlinear associations with PA outcomes. ML models using all variables achieved excellent predictive performance, including AUC = 0.993, accuracy=0.981, sensitivity=0.954, specificity=0.990, PPV = 0.966, NPV = 0.986, F1-score=0.960 and Youden’s index=0.944. After restricting predictors to regression-identified variables, the CatBoost model maintained acceptable discrimination, with an AUC = 0.754, accuracy=0.785, sensitivity=0.342, specificity=0.925, PPV = 0.621, NPV = 0.823, F1-score =0.441 and Youden’s index=0.279. SHAP analysis consistently ranked *H.pylori* infection as the top predictor. Longitudinal analysis revealed a higher proportion of persistent *H.pylori* infection in emerging PA cases. Mediation analysis indicated a negligible indirect effect of the TyG index (1.11%, *P* = 0.124).

**Conclusions:**

*H.pylori* infection is independently associated with PA and represents a critical contributor to PA risk stratification. The integration of epidemiological analysis with interpretable ML provides a robust framework for identifying high-risk individuals and supports the potential value of incorporating infectious markers into vascular risk assessment.

## Introduction

Peripheral atherosclerosis (PA), clinically manifested as peripheral artery disease (PAD), is a systemic atherosclerotic disorder characterized by progressive narrowing and occlusion of peripheral arteries, most commonly affecting the lower extremities (Narula et al., 2018). PA represents a major global public health challenge, affecting more than 200 million individuals worldwide and contributing substantially to cardiovascular morbidity and mortality (Song et al., 2019b). Individuals with PA face a markedly increased risk of myocardial infarction, stroke, major adverse cardiovascular events (MACE), major adverse limb events (MALE), and all-cause mortality compared with those without the disease (Kokkinidis et al., 2020). In China, the burden of PA has risen steadily over recent decades, driven by population aging and the increasing prevalence of metabolic risk factors, yet the condition remains underdiagnosed, particularly in its early and asymptomatic stages (Song et al., 2019a). While traditional risk factors such as smoking, diabetes, dyslipidemia, and hypertension are well established, growing evidence suggests that chronic inflammation and infectious exposures may also play important roles in PA pathogenesis (Rosenfeld and Campbell, 2011). Although Helicobacter pylori (*H. pylori*) infection has been linked to systemic atherosclerosis and cardiovascular disease (Xu et al., 2017; Sun et al., 2023), its association with PA remains insufficiently explored and yields inconsistent findings, especially in large population-based studies. This gap highlights the need for further investigation into the potential relationship between *H. pylori* infection and peripheral atherosclerosis.

*H. pylori* infection has been increasingly implicated as a potential contributor to peripheral atherosclerosis through its systemic proinflammatory and metabolic effects. *H. pylori* infection has been associated with elevated circulating inflammatory markers, including high-sensitivity C-reactive protein (hs-CRP), which is a recognized predictor of atherosclerotic cardiovascular risk (Wang et al., 2020). Persistent low-grade inflammation induced by *H. pylori* may promote endothelial dysfunction, oxidative stress, and subsequent atherogenesis (Temesgen et al., 2022). In addition, several studies have reported associations between *H. pylori* infection and adverse lipid profiles, characterized by increased low-density lipoprotein cholesterol and reduced high-density lipoprotein cholesterol, both of which are key drivers of atherosclerotic disease progression (Kim et al., 2020; Fallah et al., 2021). Emerging evidence further suggests that *H. pylori* may exacerbate atherosclerosis by inducing insulin resistance, a central metabolic abnormality linking inflammation to vascular damage (Azami et al., 2021). The triglyceride-glucose (TyG) index, a validated surrogate marker of insulin resistance, has therefore gained attention as a potential mediator in the relationship between *H. pylori* infection and vascular disease (Khan et al., 2018). However, existing studies have largely focused on coronary or carotid atherosclerosis, relied on limited sample sizes, or lacked comprehensive adjustment for metabolic confounders, leaving the association between *H. pylori* infection and peripheral atherosclerosis insufficiently characterized. These limitations underscore the need for large-scale, well-controlled studies to clarify the role of *H. pylori* in PA.

Machine learning (ML) provides a powerful analytical framework for identifying complex, nonlinear relationships among multiple clinical and biochemical variables that may not be adequately captured by traditional statistical methods (Bastanlar and Ozuysal, 2014; Zhu et al., 2024; Zheng et al., 2025). In the context of peripheral atherosclerosis, ML is particularly suitable for integrating heterogeneous risk factors, including infectious status, metabolic indicators, and inflammatory markers, to improve risk stratification and disease prediction (Munger et al., 2021). Compared with deep learning approaches, classical ML algorithms are more appropriate for structured clinical datasets of moderate sample size, such as those derived from routine health examinations, as they require fewer parameters, are less prone to overfitting, and offer greater model stability and interpretability (Amirgaliyev et al., 2025). Previous studies have successfully applied ML techniques to predict PAD-related outcomes, including hospitalization risk (Berger et al., 2020), postoperative complications (Kerasidou, 2020), and disease progression (Chang et al., 2020), underscoring their potential utility in vascular medicine. Importantly, the incorporation of interpretable ML methods, such as SHapley Additive exPlanations (SHAP), enables transparent quantification of individual feature contributions, thereby facilitating biological interpretation and clinical trust (Chen et al., 2023). From a practical perspective, ML models combined with SHAP analysis can support early identification of individuals at high risk for peripheral atherosclerosis, inform targeted screening strategies, and assist clinicians in personalized risk assessment by highlighting modifiable factors such as *H. pylori* infection and metabolic dysfunction (Lundberg and Lee, 2017; Liu et al., 2024). However, the application of interpretable ML approaches to systematically evaluate the relationship between *H. pylori* infection and peripheral atherosclerosis remains limited, highlighting a critical gap that the present study seeks to address.

Despite increasing concern regarding the links between *H. pylori* and cardiovascular diseases, its relationship with PAD remains controversial (Shi et al., 2022). Most existing studies have relied on cross-sectional designs, which limit causal inference and provide only preliminary insights into this association (Sawayama et al., 2008). In this context, the present study was designed with two primary objectives. On the one hand, we aimed to systematically investigate the association between *H. pylori* infection and PA in a large, population-based Chinese cohort by integrating conventional epidemiological analyses with comprehensive adjustment for demographic, metabolic, and inflammatory confounders. Moreover, we sought to develop and validate machine learning-based predictive models for PA, using a range of routinely available clinical and biochemical indicators, including *H. pylori* infection status, lipid profiles, inflammatory markers, and metabolic indices such as the triglyceride-glucose index. Multiple ML algorithms were applied and compared to evaluate predictive performance, and model robustness was assessed using standard accuracy metrics. To enhance interpretability and clinical relevance, SHAP were employed to quantify and visualize the contribution of individual predictors across different models, thereby identifying key risk factors and illustrating their consistency and reliability. Through this integrative approach, our study aims to clarify the role of *H. pylori* infection in PA and to demonstrate the potential of interpretable ML for risk stratification and early identification of individuals at high risk for PA.

## Materials and methods

### Study participants

This study included individuals who underwent routine health examinations at The Affiliated Yueqing Hospital of Wenzhou Medical University between January 2018 and December 2024. The participant selection process is illustrated in **Supplementary Figure 1**. Initially, a total of 6,170 individuals with *H. pylori* detection were screened during the study period. Among them, 33 participants were excluded due to a history of gastrointestinal surgery, 16 due to pregnancy, 78 due to activated thyroid disorders, 68 due to severe cardiovascular disease, 28 due to history of malignant tumors, and 88 due to incomplete clinical data. After applying these exclusion criteria, 5,862 participants were finally included for the subsequent analysis. Eligible participants had complete clinical data, including demographic characteristics (age and sex), lifestyle factors (smoking and alcohol consumption), blood pressure measurements, laboratory parameters, urea breath test results for *H. pylori* infection, and lower-extremity vascular ultrasound findings. All participants underwent repeated health examinations, with a minimum interval of six months between the initial and final assessments. This study was approved by the Ethics Committee of The Affiliated Yueqing Hospital of Wenzhou Medical University (Approval No. YQYY202500055), and all procedures were conducted in accordance with the Declaration of Helsinki.

### Peripheral atherosclerosis measurement

All participants underwent bilateral lower extremity arterial assessments using two-dimensional B-mode ultrasound with a scanning frequency from 7 to 12 MHz (Siemens AG, ACUSON S2000). Multiple vascular parameters were detected by an experienced sonographer to determine the extent of arterial stenosis and atherosclerosis burden. These parameters included vascular lumen, intima-media thickness (IMT), blood flow velocity, and resistance index (RI). Peripheral atherosclerosis was defined as the presence of arterial segments with an IMT greater than 1.0 mm or exceeding 50% of the thickness of adjacent segments (Ibañez et al., 2009).

### *H. pylori* infection detection

*H. pylori* infection was assessed using either the ^13^C or ^14^C urea breath tests (UBT) (Fischbach and Malfertheiner, 2018). For the ^13^C-UBT, participants were instructed to fast before the test, after which a baseline breath sample was collected. Subsequently, a ^13^C-urea capsule was administered, and a second breath sample was taken 30 minutes later. Both samples were analyzed using specialized diagnostic equipment with Delta over Baseline (DOB) value ≥4% as positive *H. pylori* infection. In the case of the ^14^C-UBT, individuals ingested a ^14^C-urea capsule with water, waited for approximately 15 minutes, and then exhaled slowly into a collection device for 1–3 minutes. The resulting breath sample was analyzed by inserting the gas collection card into a dedicated detection instrument.

### Clinical data collection

Comprehensive clinical data were collected from all participants and categorized into demographic information, lifestyle factors, clinical examinations, and laboratory measurements. Demographic information included age and sex. Lifestyle factors comprised smoking status and alcohol consumption history, which are established contributors to atherosclerotic risk. Clinical examination data included seated measurements of systolic (SBP) and diastolic blood pressure (DBP), reflecting hemodynamic status and vascular load. After an overnight fast of at least 8 hours, venous blood samples were obtained to assess laboratory parameters related to metabolic, lipid, and inflammatory status, including white blood cell (WBC), hemoglobin (HB), platelet (PLT), C-reactive protein (CRP), fasting blood glucose (FBG), glycated hemoglobin A1c (HbA1c), triglycerides (TG), total cholesterol (TC), low-density lipoprotein (LDL), high-density lipoprotein (HDL), and serum ferritin (SF). All laboratory assessments and vascular ultrasound examinations were performed on the same day to ensure consistency. In addition, the triglyceride-glucose (TyG) index, a validated surrogate marker of insulin resistance, was calculated using the formula: ln[TG (mg/dL) x FBG (mg/dL)/2] (Guerrero-Romero et al., 2010). Collectively, these clinical data were incorporated into subsequent analyses to evaluate traditional and metabolic risk factors and to explore their potential contributions to the development of peripheral atherosclerosis.

### ML algorithms and model evaluation

To preliminarily identify clinical factors associated with PA, univariate logistic regression analyses were first performed for all candidate demographic, lifestyle, and laboratory variables, followed by multivariate logistic regression to adjust for potential confounders. RCS analyses and subgroup analyses were further conducted to explore nonlinear associations and effect heterogeneity. Subsequently, the full cohort was randomly divided into a training set (70%) and an internal validation set (30%). In the initial modeling stage, all available clinical variables were incorporated into ML models to evaluate overall predictive performance. A comprehensive set of ML algorithms was implemented in accordance with the scikit-learn user guide (https://scikit-learn.org/stable/user_guide.html), including Logistic Regression, Lasso Regression, Support Vector Machine, K-Nearest Neighbors, Partial Least Squares, Linear and Quadratic Discriminant Analysis, Gaussian Naïve Bayes, Multi-layer Perceptron (MLP), Random Forest, Gradient Boosting Decision Tree, AdaBoost, Extreme Gradient Boosting (XGBoost), CatBoost, and Light Gradient Boosting Machine (LightGBM). These models were selected to represent a range of linear, nonlinear, tree-based, ensemble, and neural network approaches. Model performance was evaluated using metrics consistent with those reported in the abstract, including area under the area under curve (AUC), accuracy, sensitivity, specificity, positive predictive value (PPV), negative predictive value (NPV), F1 score, and Youden’s index. Following the identification of significant predictors based on logistic regression, RCS, and subgroup analyses, a second-stage ML modeling process was conducted using the reduced feature set. Model performance metrics before and after feature reduction were compared to assess the robustness of the predictive models and to evaluate whether the feature selection process preserved discriminative ability. Finally, the diagnostic utility of the optimal model was assessed through comprehensive evaluations, comprising of decision curve analysis (DCA), reverse cumulative residual distribution, Neural Network prediction, and Calibration curve analysis.

### SHAP interpretation

To evaluate the contribution of each clinical variable to the predictive performance of the PA model, we calculated the SHAP values derived from cooperative game theory using the “shapviz” R package. Due to its limited application only on XGBoost and LightGBM models, we displayed the importance ranking for each clinical variable based the optimal predictive model (Singh et al., 2017). SHAP values provide an interpretable framework to quantify the marginal impact of each feature on model output, offering transparency for complex machine learning algorithms. Subsequently, we employed multiple SHAP-based visualization techniques to comprehensively illustrate the relative importance and influence of individual features within the optimal ML model.

### Statistical analysis

Continuous variables were analyzed using independent sample t-tests or Wilcoxon tests and expressed as means ± standard deviations. Categorical data were assessed via chi-square tests and summarized as frequencies and percentages. To explore longitudinal changes, participants were categorized according to *H. pylori* infection status at baseline and follow-up into four groups: persistent negative, recovery infection, emerging infection, and persistent infection. Peripheral atherosclerosis status was similarly classified into persistent PA, emerging PA, and persistent negative PA. Differences in *H. pylori* infection trajectories between PA-positive and PA-negative subgroups were compared, and intergroup differences in triglyceride-glucose (TyG) index levels were evaluated across longitudinal *H. pylori* status categories. RCS models were applied to assess potential nonlinear associations between the TyG index, *H. pylori* infection status, and PA outcomes. To further examine whether the TyG index mediated the association between *H. pylori* infection and PA, formal mediation analysis was performed using the “mediation” package in R. Logistic regression models were built with the “finalfit” package, while RCS analyses were carried out using the “foreign” and “rms” packages in R. Machine learning model development and validation were implemented with involved multiple R packages, including “caret”, “kernelshap”, “rmda”, “catboost”, “lightgbm”, and “DALEX”. SHAP values were calculated utilizing the “shapviz” R package to interpret model outputs. All statistical analyses were performed using R (version 4.1.3). A two-sided p-value <0.05 after false discovery rate (FDR) adjustment was considered statistically significant.

## Results

### Baseline clinical features of participants

This study included 5,862 individuals with a mean age of 49.94 years, comprising of 3,351 *H. pylori*-negative and 2,511 *H. pylori*-positive participants. The cohort consisted of 3,988 males (68.03%) and 1,874 females (31.97%), with an overall *H. pylori* positivity rate of 42.84%. Compared to *H. pylori*-negative individuals, *H. pylori*-positive cohorts exhibited higher proportions of age ≥60 years, smoking, and peripheral atherosclerosis, as well as higher levels of HbA1c. The detailed demographic and clinical characteristics of subjects was summarized in **Supplementary Table 1**.

### Clinical difference between peripheral atherosclerosis subgroups

The chi-square tests and either t-tests or Wilcoxon tests were employed to initially investigate the clinical characteristics associated with PA patients. Compared to PA-negative individuals, those with PA exhibited significantly higher proportions of classical risk features, including advanced age (51.73% vs 4.33%), male (86.42% vs 62.50%), smoking (50.11% vs 25.45%), and alcohol consumption (64.21% vs 16.15%). In addition, patients with PA had elevated levels of CRP, TC, LDL, SBP, DBP, FBG, and HbA1c (*P* <0.001), which is consistent with previously reported findings. Notably, *H. pylori*-positive cohorts occupied a higher prevalence in PA patients (57.71% vs 38.36%), indicating a potential connection between *H. pylori* infection and the development of PA (**Table 1**).

**Table 1:** The clinical characteristics of peripheral atherosclerosis subgroups.

Variables	PA-negative (n=4507)	PA-positive (n=1355)	*P*-value
Age (n, %)			<0.001***
≥60	195 (4.33%)	701 (51.73%)	
<60	4312 (95.67%)	654 (48.27%)	
Gender (n, %)			<0.001***
Male	2817 (62.50%)	1171 (86.42%)	
Female	1690 (37.50%)	184 (13.58%)	
Smoke (n, %)			<0.001***
Yes	1147 (25.45%)	679 (50.11%)	
No	3360 (74.55%)	676 (49.89%)	
Drink (n, %)			<0.001***
Yes	728 (16.15%)	870 (64.21%)	
No	3779 (83.85%)	485 (35.79%)	
White blood cell (10^9/L)	5.55 ± 0.65	5.57 ± 0.64	0.379
Haemoglobin (g/L)	132.29 ± 2.55	132.36 ± 2.64	0.408
Platelet (10^9/L)	167.55 ± 19.30	167.77 ± 19.14	0.709
C-reactive protein (mg/L)	10.31 ± 1.98	10.98 ± 2.17	<0.001***
Triglycerides (mmol/L)	1.96 ± 1.69	2.05 ± 1.66	0.096
Total cholesterol (mmol/L)	5.01 ± 0.93	5.22 ± 0.99	<0.001***
High density lipoprotein (mmol/L)	1.38 ± 0.29	1.39 ± 0.28	0.233
Low density lipoprotein (mmol/L)	2.67 ± 0.69	2.82 ± 0.73	<0.001***
Systolic blood pressure (mm/Hg)	126.03 ± 16.62	135.05 ± 18.51	<0.001***
Diastolic blood pressure (mm/Hg)	76.62 ± 11.67	79.89 ± 11.00	<0.001***
Fasting blood glucose (mmol/L)	5.36 ± 1.40	5.78 ± 1.76	<0.001***
Glycosylated hemoglobin A1c (%)	5.84 ± 0.85	6.12 ± 1.08	<0.001***
Serum ferritin (μg/L)	612.64 ± 309.70	598.76 ± 299.43	0.145
H. pylori infection (n, %)			<0.001***
Yes	1729 (38.36%)	782 (57.71%)	
No	2778 (61.64%)	573 (42.29%)	

***p.value <0.001. PA, peripheral atherosclerosis; H. pylori, Helicobacter pylori. The bold values means statistical significance.

### Univariate and multivariate logistic regression analysis of risk factors for peripheral atherosclerosis

**Figure 1:**
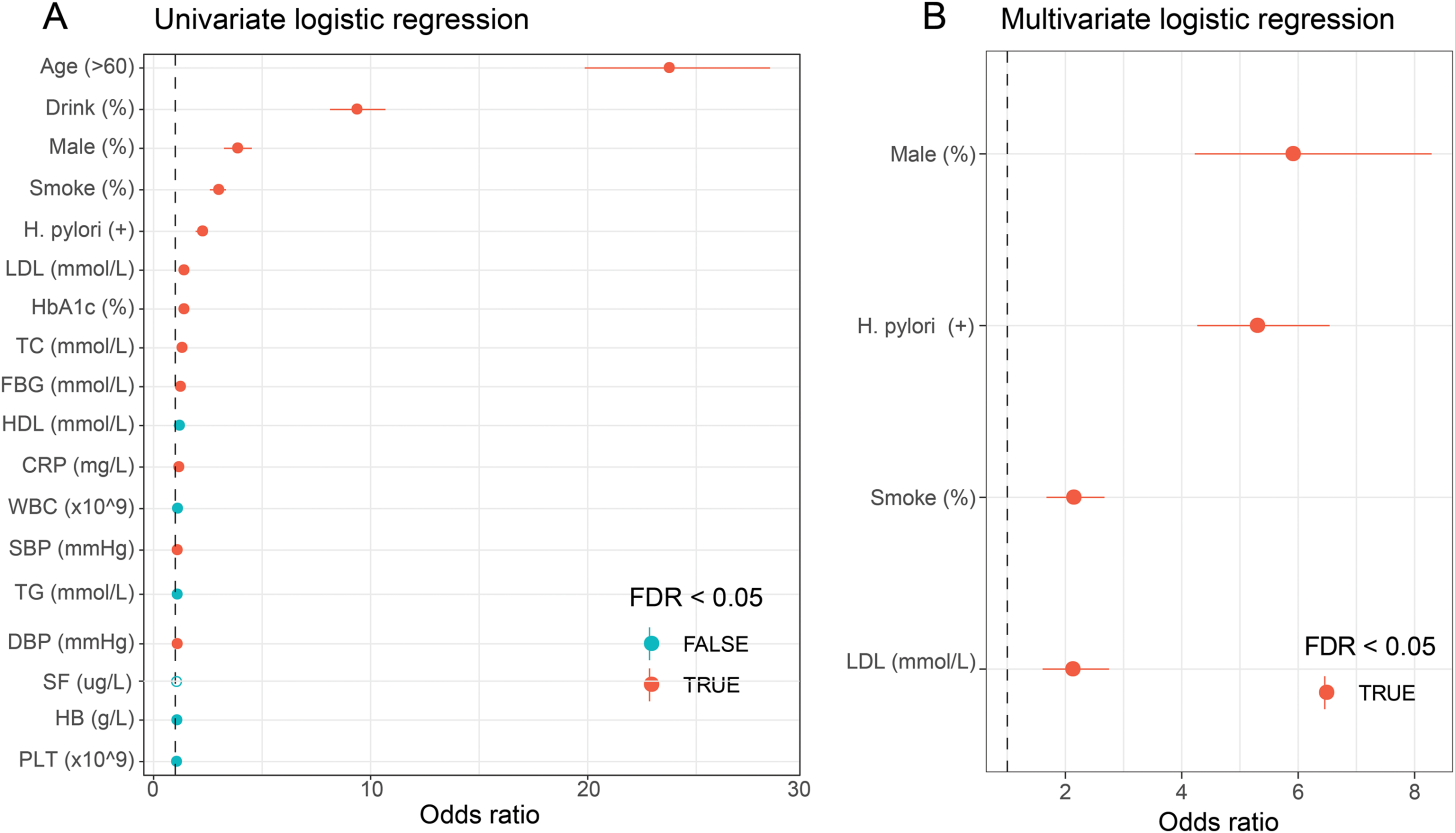
Univariate **(A)** and Multivariate **(B)** logistic regression analysis of risk factors for peripheral atherosclerosis.

Univariate logistic regression analysis demonstrated that *H. pylori* infection was significantly associated with an increased risk of PA (OR = 2.19, 95% CI = 1.94-2.48, *P* <0.001). In addition, other variables, such as age >60, alcohol consumption, male, smoking, elevated levels of CRP, LDL, HbA1c, TC, FBG, SBP, and DBP, were also identified as significant risk factors for PA (**Figure 1A**, **Supplementary Table 2**). Following multivariate stepwise regression analysis, *H. pylori* infection remained an independent and robust risk factor for peripheral atherosclerosis (OR =5.27, 95%CI =4.27-6.54, *P* <0.001), along with male (OR =5.88, 95%CI=4.22-8.29, *P* <0.001), smoking (OR =2.11, 95%CI=1.67-2.67, *P* <0.001), and LDL levels (OR =2.10, 95%CI=1.47-3.04, *P* <0.001) (**Figure 1B**, **Supplementary Table 3**).

### Subgroup analysis and RCS analysis

To further validate the stability of critical clinical parameters, we performed the RCS and subgroup analyses to explore nonlinear associations and effect heterogeneity. The subgroup analysis indicated that emerging and persistent PA patients exhibited higher proportions of advanced age, male, smoking, and alcohol consumption, as well as elevated levels of WBC, PLT, CRP, TC, LDL, SBP, DBP, FBG, and HbA1c (*P* <0.05, **Supplementary Table 3**). Moreover, the RCS analysis revealed a significantly nonlinear relationship between multiple clinical parameters and PA status in *H. pylori* positive cohorts, including CRP, TG, TC, LDL, HbA1c, FBG, DBP and SBP (**Supplementary Figure 2**). Therefore, the coincident risk factors (*H. pylori* infection, male, smoking and LDL) among multivariate logistic regression, subgroup analysis and RCS analysis were ultimately identified for ML model’s construction.

### Screening optimal ML models for peripheral atherosclerosis prediction

**Figure 2:**
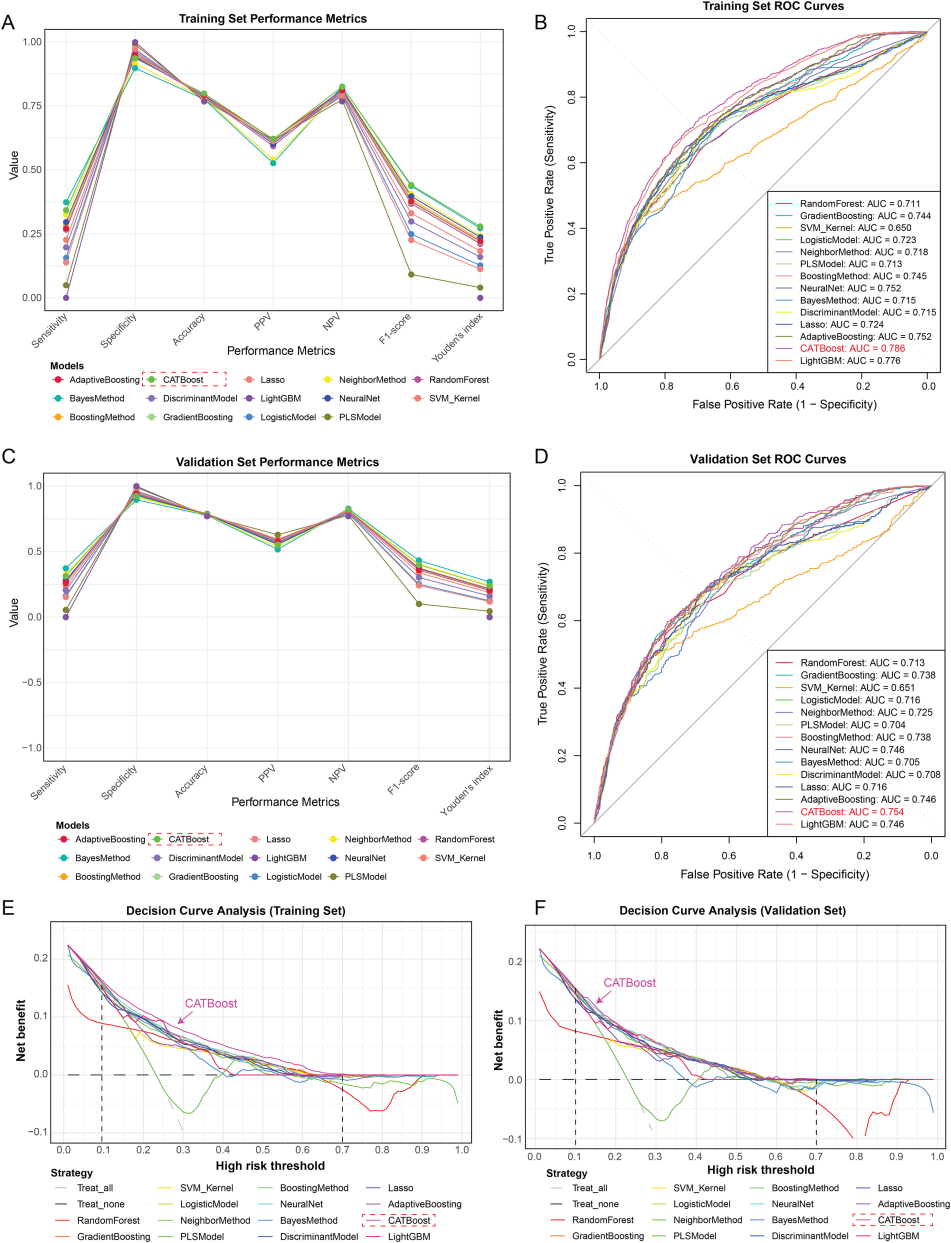
Screening optimal ML models for peripheral atherosclerosis prediction. The distribution of model’s metrics from 14 Machine learning algorithms in Training **(A)** and Validation sets **(C)**, including sensitivity, accuracy, specificity, PPV, NPV, F1-score, and Youden’s index. The ROC curves of 14 Machine learning models in Training **(B)** and Validation sets **(D)**. Clinical decision analysis indicated that the majority of ML models (except PLS model) improved the potential clinical benefits for PA risk prediction in both Training **(E)** and Validation sets **(F)**.

To further construct an optimal diagnostic model for PA, we respectively employed 14 distinct ML algorithms based on all and critical clinical parameters from the multivariate regression analysis. Considering all performance metrics, the CATBoost model exhibited the most favorable predictive performance using all clinical parameters. The CATBoost model achieved an AUC of 0.993, accuracy of 0.981, Sensitivity of 0.954, specificity of 0.990, PPV of 0.966, NPV of 0.986, F1-score of 0.960 and Youden’s index of 0.944 (**Supplementary Table 5**). Using the critical clinical parameters, this model still remained superior predictive ability in both Training and Validation sets. In Training sets, the CATBoost model achieved an AUC of 0.786, with an accuracy of 0.799, specificity of 0.937, PPV of 0.621, NPV of 0.825, F1-score of 0.441, and Youden’s index of 0.279 (**Figures 2A, B**; **Supplementary Table 6**). Consistently, in Validation sets, the CATBoost model maintained robust performance, with an AUC of 0.754, accuracy of 0.785, specificity of 0.925, PPV of 0.553, NPV of 0.819, F1-score of 0.399, and Youden’s index of 0.237 (**Figures 2C, D**; **Supplementary Table 7**). Overall, CATBoost outperformed other models across multiple evaluation metrics, making it the most effective and stable classifier for predicting PA in this study.

### Model’s diagnostic efficacy evaluation for peripheral atherosclerosis prediction

**Figure 3:**
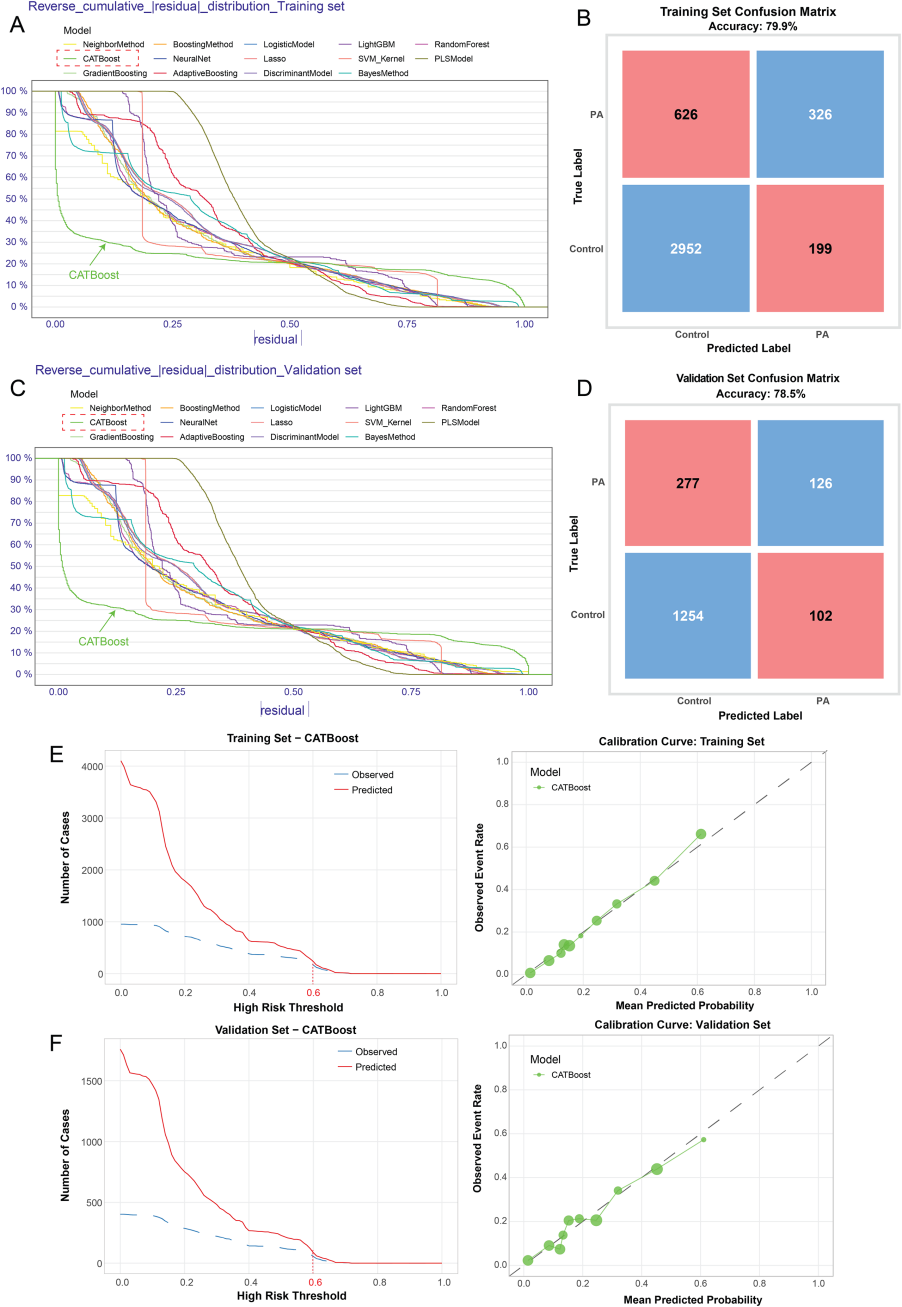
Model’s diagnostic efficacy evaluation for peripheral atherosclerosis prediction. Residual distribution analysis further revealed that the CATBoost model exhibited the lowest residual spread among all models in Training **(A)** and Validation sets **(C)**. Neural Network analysis demonstrated that CATBoost model achieved the highest predictive accuracy, with 79.9% in the training set **(B)** and 78.5% in the validation set **(D)**. Calibration curve analysis also demonstrated that predictions closely aligned with observed outcomes **(E, F)**.

DCA analysis indicated that the majority of ML models (except PLS model) based on *H. pylori* infection, male, smoking and LDL, improved the potential clinical benefits for PA risk prediction within a threshold probability range of 0.1 to 0.7 in both Training and Validation sets (**Figures 2E, F**). Residual distribution analysis further revealed that the CATBoost model exhibited the lowest residual spread among all models in both datasets, indicating superior model stability (**Figures 3A, C**). Furthermore, Neural Network analysis demonstrated that CATBoost model achieved the highest predictive accuracy, with 79.9% in the training set and 78.5% in the validation set (**Figures 3B, D**). Calibration curve analysis also demonstrated that predictions with a high-risk threshold (>0.6) closely aligned with observed outcomes, confirming the model’s calibration and reliability in both cohorts (**Figures 3E, F**). Collectively, these findings underscore the CATBoost model as the most robust and clinically advantageous predictive tool for PA, offering superior stability, calibration, and diagnostic accuracy across datasets.

### SHAP interpretation for role of *H. pylori* infection in peripheral atherosclerosis

**Figure 4:**
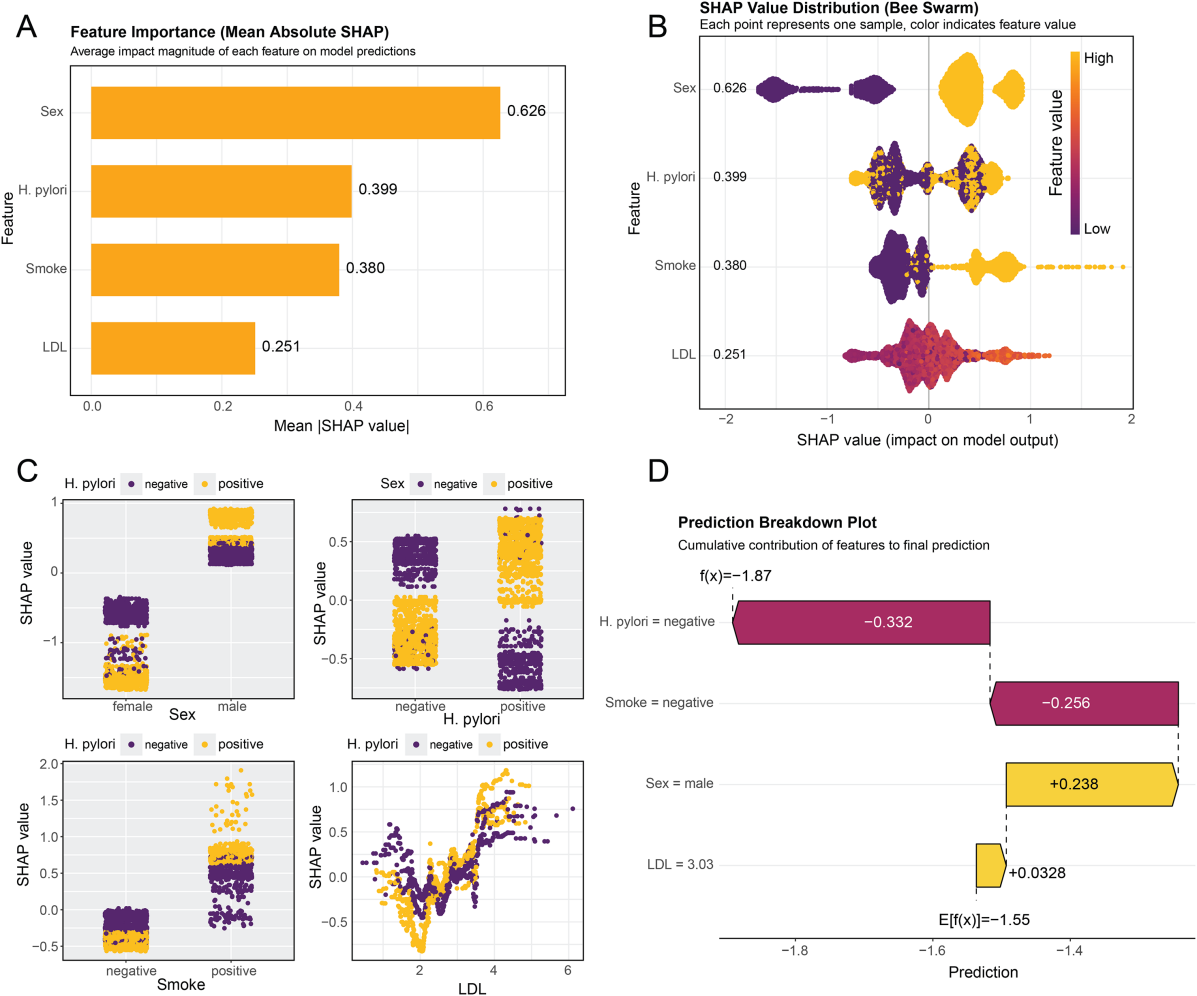
SHAP interpretation for role of H. pylori infection in peripheral atherosclerosis. The distribution of absolute SHAP values of four vital features in the CATBoost model **(A, B)**. Hierarchical comparisons of SHAP values further indicated potential interactions between H. pylori infection and other clinical variables **(C)**. Breakdown Plot from random sampling demonstrated that patients with negative H. pylori status and non-smoking history had reduced SHAP values **(D)**.

To further elucidate the role of *H. pylori* infection in the CATBoost model for predicting peripheral atherosclerosis, SHAP analysis was employed to enhance interpretability through comprehensive global and local visualizations. Using all clinical parameters in the optimal model, *H. pylori* infection exhibited excellent separating capacity second to only age and gender (**Supplementary Figure 3**). Furthermore, based on critical parameters in the predictive model, the SHAP summary plots revealed that *H. pylori* infection was the second most influential variable in the model, following gender, based on mean absolute SHAP values (**Figures 4A, B**). This highlights its substantial contribution to the model’s predictive power. Hierarchical comparisons of SHAP values further indicated potential interactions between *H. pylori* infection and other clinical variables, suggesting synergistic effects that may influence PA risk (**Figure 4C**). Moreover, individual-level SHAP force plots from random sampling demonstrated that patients with negative *H. pylori* status and non-smoking history had reduced SHAP values, indicating a protective profile against PA (**Figure 4D**). These findings suggest that besides conventional clinical parameters, *H. pylori* infection serves as a key determinant in the machine learning-based prediction of PA and warrants further investigation as a modifiable clinical marker.

### The longitudinal association between *H. pylori* infection and peripheral atherosclerosis

**Figure 5:**
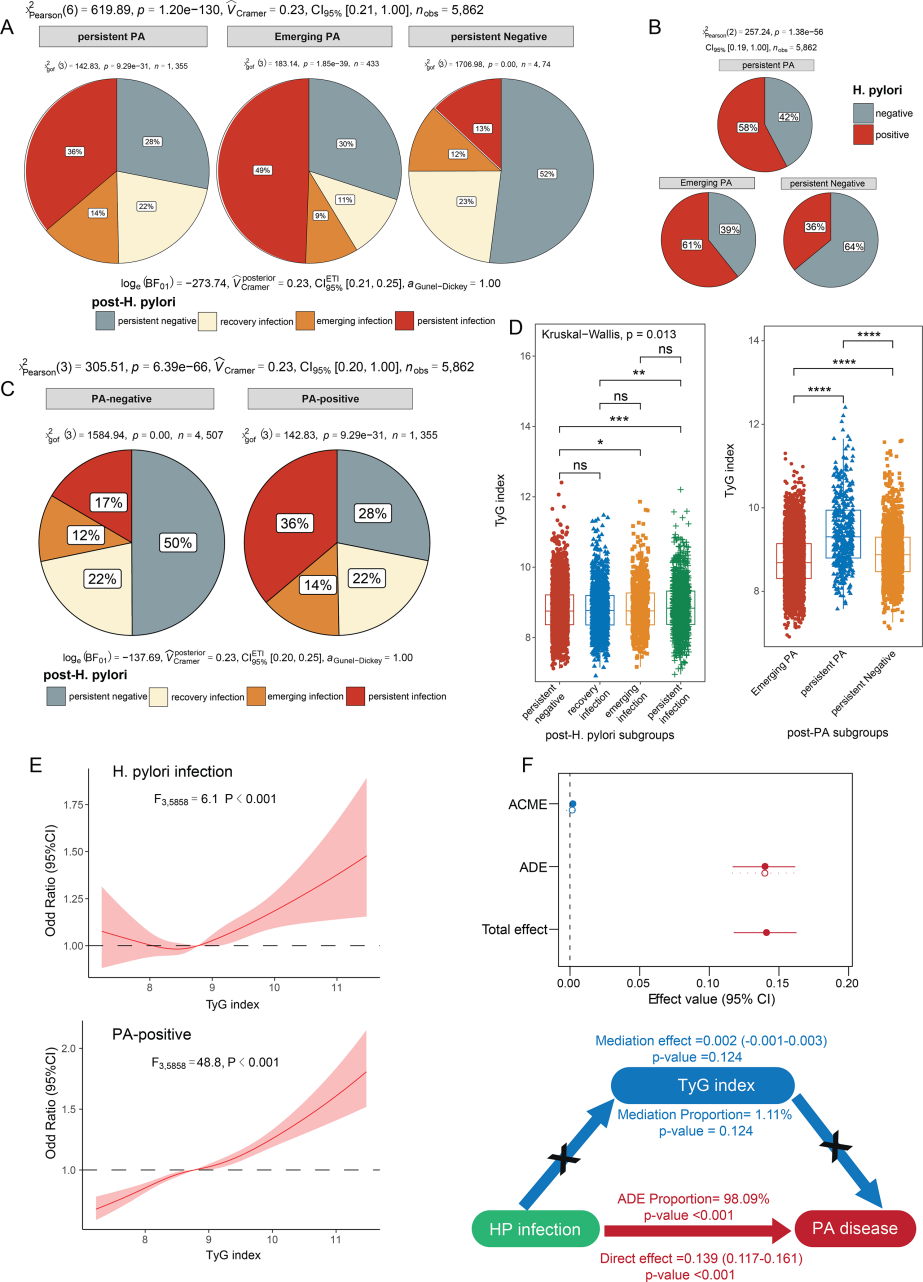
The longitudinal association and Mediation analysis among H. pylori infection, TyG index, and peripheral atherosclerosis. **(A, B)**. Comparison of initial and post-H. pylori infection among different post-PA status. **(C)**. Comparison of post-H. pylori infection among different initial PA subgroups. **(D)**. Comparison of TyG index levels among different post-H. pylori infection and post-PA status. **(E)**. The Restricted Cubic Spline analysis revealed a significantly nonlinear relationship between the TyG index and H. pylori infection, as well as positive PA status. **(F)**. Mediation analysis among H. pylori infection, TyG index, and peripheral atherosclerosis. *, P<0.05, **, P<0.01, ***, P<0.001, ****P <0.0001.

To investigate the impact of *H. pylori* infection on the development of PA, we analyzed the distribution of longitudinal *H. pylori* infection status across different PA subgroups. Persistent PA-negative individuals exhibited a lower prevalence of *H. pylori* infection at the initial examination, and were more likely to maintain a persistently negative *H. pylori* status by the final follow-up. In contrast, the emerging PA subgroups demonstrated the highest proportion of persistent *H. pylori* infection, suggesting a potential association between sustained infection and the onset of PA (**Figures 5A, B**). Furthermore, compared to PA-negative groups, PA-positive patients showed a significantly higher proportion of persistent *H. pylori* infection and a markedly lower rate of persistently negative *H. pylori* status (**Figure 5C**).

### Mediation analysis among *H. pylori* infection, TyG index, and peripheral atherosclerosis

The TyG index has been widely recognized as a high-risk factor for PA. The elevated TyG index levels were not only observed in persistent PA subgroups, but also among those with emerging and persistent *H. pylori* infection (**Figure 5D**), suggesting a potential mechanistic link involving TyG in the interplay between *H. pylori* infection and PA. The RCS analysis further revealed a significantly nonlinear relationship between the TyG index and *H. pylori* infection (F = 6.1, P <0.001), as well as positive PA status (F = 48.8, P <0.001) (**Figure 5E**). To further investigate whether the TyG index mediates the relationship between *H. pylori* infection and PA, a mediation analysis was performed. The results indicated that TyG index didn’t mediate the relationship between *H. pylori* and PA (mediation proportion =1.11%, p-value =0.124). Contrastively, *H. pylori* infection exerted a strong direct effect on PA, with an average direct effect (ADE) proportion =98.09%, p-value <0.001 (**Figure 5F**). These findings highlight the predominant direct impact of *H. pylori* infection on PA, independent of the TyG index.

## Discussion

Vascular disease remains the foremost cause of mortality around the world, with a yearly increase in cases seen in both developed and developing regions (Mensah et al., 2019). The common condition of atherosclerosis underlies cardiovascular disease and peripheral artery diseases, featuring a damaged endothelium, chronic low-level inflammation, lipid deposits, and plaque formation in the inner layer of blood vessels (Tsao et al., 2022). While traditional risk factors such as smoking, hypertension, dyslipidemia, and diabetes explain a large share of disease burden, they do not fully account for interindividual variability in peripheral PA (Kennedy et al., 2005). Growing interest has therefore focused on nontraditional contributors, including chronic infection and systemic inflammation. Emerging evidences highlighted a strong link between *H. pylori* infection and cardiovascular incidents, indicating that the infection can triple or quadruple the risk of these events (Nam et al., 2015). However, the association between *H. pylori* and PA has remained insufficiently characterized, motivating the present investigation.

In this large population-based study, we systematically investigated the association between *H. pylori* infection and PA using an interpretable ML framework. Our findings demonstrate that *H. pylori* infection is significantly associated with an increased risk of PA and consistently emerged as an important predictor across multiple ML models. In addition, metabolic factors, particularly the TyG index, were identified as key contributors to PA risk. These results suggest that *H. pylori* infection is not merely a localized gastrointestinal condition but may exert broader systemic effects that contribute to peripheral vascular pathology. By integrating traditional epidemiological analyses with explainable ML techniques, our study provides robust and complementary evidence supporting a link between infectious, metabolic, and vascular processes in PA.

Our findings are broadly consistent with previous studies reporting associations between *H. pylori* infection and cardiovascular diseases. Several epidemiological investigations have shown that *H. pylori* infection is associated with an increased risk of coronary artery disease and ischemic stroke, potentially mediated by systemic inflammation and endothelial dysfunction (Oshima et al., 2005; Moutsopoulos and Madianos, 2006). For example, Mohamed et al (Riad, 2021). reported a significant association between *H. pylori* infection and coronary artery disease, while Baye et al (Baye et al., 2024). observed elevated inflammatory markers and adverse lipid profiles in *H. pylori*-positive individuals. These observations align with our results showing higher LDL levels and increased PA risk among infected participants.

However, evidence specifically linking *H. pylori* infection to PA remains limited and inconsistent. Some earlier studies failed to identify a clear association, possibly due to smaller sample sizes, heterogeneous diagnostic criteria for PA, or insufficient adjustment for metabolic confounders. Our study extends the existing literature by focusing specifically on PA, incorporating a large Chinese cohort, and applying ML models capable of capturing nonlinear interactions among multiple risk factors. Moreover, the identification of the TyG index as an important predictor supports prior research highlighting insulin resistance as a central mechanism in atherosclerosis progression (Cubbon et al., 2012; Brie et al., 2025). The co-identification of *H. pylori* and TyG index in our analysis supports the theory that infectious and metabolic factors may synergistically accelerate atherosclerotic processes.

Our findings are also supported by mechanistic studies. *H. pylori* infection has been shown to increase levels of inflammatory markers such as interleukin-6, tumor necrosis factor-alpha, and hs-CRP (Crabtree et al., 1991; Begolli-Stavileci et al., 2020). These cytokines induce endothelial dysfunction, promote LDL oxidation, and enhance monocyte adhesion, all of which are critical steps in the development of atherosclerosis (Peluso et al., 2012; Jiang et al., 2022). Furthermore, *H. pylori* infection is associated with lipid metabolism disruption, insulin resistance, and oxidative stress-all well-established contributors to vascular damage (Cherkas et al., 2016; Ye et al., 2023). Notably, the Mediation analysis indicated that the effect of *H. pylori* infection on PA was primarily induced by direct effects rather than mediation role from TyG index, suggesting its independent influence on PA.

The clinical implications of our findings are substantial. Early identification of individuals at high risk for PA remains challenging due to the common asymptomatic nature of the disease in its early stages. Our results suggest that *H. pylori* infection status, when combined with metabolic and inflammatory indicators, may improve PA risk stratification. From a clinical perspective, this raises the possibility that screening for *H. pylori* infection could contribute to vascular risk assessment, particularly in populations with a high prevalence of metabolic disorders. From a methodological standpoint, this study highlights the value of interpretable ML approaches in cardiovascular research. By integrating SHAP with multiple ML models, we were able not only to achieve reliable predictive performance but also to transparently quantify the contribution of individual risk factors. This approach improves upon traditional regression-based analyses by accounting for complex, non-linear interactions between variables and offering individualized explanations (Ejiyi et al., 2025). Furthermore, our study contributes to the growing body of evidence supporting the role of chronic infection and metabolic dysfunction in atherosclerosis, thereby offering new directions for future mechanistic and interventional research.

However, several limitations must be acknowledged. First, this was a single-center, cross-sectional observational study, which precludes causal inference and limits conclusions to associations rather than definitive mechanisms. Second, although a comprehensive set of confounders was adjusted for, residual confounding from unmeasured factors such as dietary habits, socioeconomic status, medication use, and physical activity cannot be excluded. Third, although the urea breath test is reliable, we did not evaluate *H. pylori* heterogeneity (e.g., CagA-positive strains), which may differ in virulence and vascular risk (Pasceri et al., 2006). Fourth, our population was limited to individuals attending health examinations at a single hospital in China, potentially limiting the generalizability to other ethnic or geographic populations. Finally, although internal validation was performed, external validation of the ML models in independent cohorts is necessary to confirm their stability and predictive utility.

## Conclusions

In conclusion, this study demonstrates that *H. pylori* infection is independently associated with PA and represents an important contributor to PA risk stratification. By integrating traditional epidemiological analyses with interpretable machine learning approaches, we established a robust and transparent framework for identifying individuals at high risk of PA. Key predictors, including male, *H. pylori* infection, smoking, and LDL cholesterol levels, were consistently highlighted across analytical methods. Although the TyG index was correlated with both *H. pylori* infection and PA, mediation analysis suggested that it did not substantially account for this indirect effect. Collectively, these findings support the potential value of incorporating infectious markers into vascular risk assessment and underscore the complementary role of interpretable machine learning in advancing cardiovascular risk prediction.

## Data Availability

The original contributions presented in the study are included in the article/**Supplementary Material**. Further inquiries can be directed to the corresponding author.

## References

[B1] Amirgaliyev B. Mussabek M. Rakhimzhanova T. Zhumadillayeva A. (2025). A review of machine learning and deep learning methods for person detection, tracking and identification, and face recognition with applications. Sensors (Basel) 25, 1410. doi: 10.3390/s25051410, PMID: 40096196 PMC11902521

[B2] Azami M. Baradaran H. R. Dehghanbanadaki H. Kohnepoushi P. Saed L. Moradkhani A. . (2021). Association of Helicobacter pylori infection with the risk of metabolic syndrome and insulin resistance: an updated systematic review and meta-analysis. Diabetol. Metab. Syndr. 13, 145. doi: 10.1186/s13098-021-00765-x, PMID: 34922625 PMC8684139

[B3] Bastanlar Y. Ozuysal M. (2014). Introduction to machine learning. Methods Mol. Biol. 1107, 105–128. doi: 10.1007/978-1-62703-748-8_7, PMID: 24272434

[B4] Baye G. Wondmneh B. Ashenef B. Jemal M. Baylie T. (2024). Serum high sensitive C-reactive protein level and its correlation with lipid profile among dyspeptic patients with or without Helicobacter pylori infection in East Gojjam zone, Ethiopia. Front. Cardiovasc. Med. 11, 1470993. doi: 10.3389/fcvm.2024.1470993, PMID: 39390988 PMC11464319

[B5] Begolli-Stavileci G. Begolli G. Begolli L. (2020). Interleukin-6, tumor necrosis factor-α, and high-sensitivity C-reactive protein in diabetic patients with helicobacter pylori in Kosovo. Open Access Macedonian J. Med. Sci. 8, 188–191. doi: 10.3889/oamjms.2020.3199

[B6] Berger J. S. Haskell L. Ting W. Lurie F. Chang S. C. Mueller L. A. . (2020). Evaluation of machine learning methodology for the prediction of healthcare resource utilization and healthcare costs in patients with critical limb ischemia-is preventive and personalized approach on the horizon? EPMA J. 11, 53–64. doi: 10.1007/s13167-019-00196-9, PMID: 32140185 PMC7028871

[B7] Brie A. D. Christodorescu R. M. Popescu R. Adam O. Tirziu A. Brie D. M. (2025). Atherosclerosis and insulin resistance: is there a link between them? Biomedicines. 13, 1291. doi: 10.3390/biomedicines13061291, PMID: 40564010 PMC12189823

[B8] Chang B. Sun Z. Peiris P. Huang E. S. Benrashid E. Dillavou E. D. (2020). Deep learning-based risk model for best management of closed groin incisions after vascular surgery. J. Surg. Res. 254, 408–416. doi: 10.1016/j.jss.2020.02.012, PMID: 32197791

[B9] Chen Q. S. Bergman O. Ziegler L. Baldassarre D. Veglia F. Tremoli E. . (2023). A machine learning based approach to identify carotid subclinical atherosclerosis endotypes. Cardiovasc. Res. 119, 2594–2606. doi: 10.1093/cvr/cvad106, PMID: 37475157 PMC10730242

[B10] Cherkas A. Eckl P. Gueraud F. Abrahamovych O. Serhiyenko V. Yatskevych O. . (2016). Helicobacter pylori in sedentary men is linked to higher heart rate, sympathetic activity, and insulin resistance but not inflammation or oxidative stress. Croat Med. J. 57, 141–149. doi: 10.3325/cmj.2016.57.141, PMID: 27106356 PMC4856189

[B11] Crabtree J. E. Shallcross T. M. Heatley R. V. Wyatt J. I. (1991). Mucosal tumour necrosis factor alpha and interleukin-6 in patients with Helicobacter pylori associated gastritis. Gut. 32, 1473–1477. doi: 10.1136/gut.32.12.1473, PMID: 1773951 PMC1379245

[B12] Cubbon R. M. Ali N. Sengupta A. Kearney M. T. (2012). Insulin- and growth factor-resistance impairs vascular regeneration in diabetes mellitus. Curr. Vasc. Pharmacol. 10, 271–284. doi: 10.2174/157016112799959305, PMID: 22239629

[B13] Ejiyi C. J. Qin Z. Ukwuoma C. C. Nneji G. U. Monday H. N. Ejiyi M. B. . (2025). Comparative performance analysis of Boruta, SHAP, and Borutashap for disease diagnosis: A study with multiple machine learning algorithms. Network. 36, 507–544. doi: 10.1080/0954898X.2024.2331506, PMID: 38511557

[B14] Fallah S. Marsche G. Mohamadinarab M. Mohassel Azadi S. Ghasri H. Fadaei R. . (2021). Impaired cholesterol efflux capacity in patients with Helicobacter pylori infection and its relation with inflammation. J. Clin. Lipidol. 15, 218–26 e1. doi: 10.1016/j.jacl.2020.11.005, PMID: 33250430

[B15] Fischbach W. Malfertheiner P. (2018). Helicobacter pylori infection. Dtsch Arztebl Int. 115, 429–436. doi: 10.3238/arztebl.2018.0429, PMID: 29999489 PMC6056709

[B16] Guerrero-Romero F. Simental-Mendia L. E. Gonzalez-Ortiz M. Martinez-Abundis E. Ramos-Zavala M. G. Hernandez-Gonzalez S. O. . (2010). The product of triglycerides and glucose, a simple measure of insulin sensitivity. Comparison with the euglycemic-hyperinsulinemic clamp. J. Clin. Endocrinol. Metab. 95, 3347–3351. doi: 10.1210/jc.2010-0288, PMID: 20484475

[B17] Ibañez B. Badimon J. J. Garcia M. J. (2009). Diagnosis of atherosclerosis by imaging. Am. J. Med. 122, S15–S25. doi: 10.1016/j.amjmed.2008.10.014, PMID: 19110084

[B18] Jiang H. Zhou Y. Nabavi S. M. Sahebkar A. Little P. J. Xu S. . (2022). Mechanisms of oxidized LDL-mediated endothelial dysfunction and its consequences for the development of atherosclerosis. Front. Cardiovasc. Med. 9, 925923. doi: 10.3389/fcvm.2022.925923, PMID: 35722128 PMC9199460

[B19] Kennedy M. Solomon C. Manolio T. A. Criqui M. H. Newman A. B. Polak J. F. . (2005). Risk factors for declining ankle-brachial index in men and women 65 years or older: the Cardiovascular Health Study. Arch. Intern. Med. 165, 1896–1902. doi: 10.1001/archinte.165.16.1896, PMID: 16157835

[B20] Kerasidou A. (2020). Artificial intelligence and the ongoing need for empathy, compassion and trust in healthcare. Bull. World Health Organ. 98, 245–250. doi: 10.2471/BLT.19.237198, PMID: 32284647 PMC7133472

[B21] Khan S. H. Sobia F. Niazi N. K. Manzoor S. M. Fazal N. Ahmad F. (2018). Metabolic clustering of risk factors: evaluation of Triglyceride-glucose index (TyG index) for evaluation of insulin resistance. Diabetol. Metab. Syndr. 10, 74. doi: 10.1186/s13098-018-0376-8, PMID: 30323862 PMC6173832

[B22] Kim D. H. Son B. K. Min K. W. Han S. K. Na J. U. Choi P. C. . (2020). Chronic gastritis is associated with a decreased high-density lipid level: histological features of gastritis based on the updated sydney system. J. Clin. Med. 9, 1856. doi: 10.3390/jcm9061856, PMID: 32545889 PMC7355915

[B23] Kokkinidis D. G. Arfaras-Melainis A. Giannopoulos S. Katsaros I. Jawaid O. Jonnalagadda A. K. . (2020). Statin therapy for reduction of cardiovascular and limb-related events in critical limb ischemia: A systematic review and meta-analysis. Vasc. Med. 25, 106–117. doi: 10.1177/1358863X19894055, PMID: 31964311

[B24] Liu L. Bi B. Cao L. Gui M. Ju F. (2024). Predictive model and risk analysis for peripheral vascular disease in type 2 diabetes mellitus patients using machine learning and shapley additive explanation. Front. Endocrinol. (Lausanne). 15, 1320335. doi: 10.3389/fendo.2024.1320335, PMID: 38481447 PMC10933094

[B25] Lundberg S. M. Lee S.-I. (2017). A unified approach to interpreting model predictions. Adv. Neural Inf. Process. Syst. 30.

[B26] Mensah G. A. Roth G. A. Fuster V. (2019). The global burden of cardiovascular diseases and risk factors: 2020 and beyond. J. Am. Coll. Cardiol. 74, 2529–2532. doi: 10.1016/j.jacc.2019.10.009, PMID: 31727292

[B27] Moutsopoulos N. M. Madianos P. N. (2006). Low-grade inflammation in chronic infectious diseases: paradigm of periodontal infections. Ann. N Y Acad. Sci. 1088, 251–264. doi: 10.1196/annals.1366.032, PMID: 17192571

[B28] Munger E. Hickey J. W. Dey A. K. Jafri M. S. Kinser J. M. Mehta N. N. (2021). Application of machine learning in understanding atherosclerosis: Emerging insights. APL Bioeng. 5, 011505. doi: 10.1063/5.0028986, PMID: 33644628 PMC7889295

[B29] Nam S. Y. Ryu K. H. Park B. J. Park S. (2015). Effects of Helicobacter pylori infection and its eradication on lipid profiles and cardiovascular diseases. Helicobacter. 20, 125–132. doi: 10.1111/hel.12182, PMID: 25382033

[B30] Narula N. Dannenberg A. J. Olin J. W. Bhatt D. L. Johnson K. W. Nadkarni G. . (2018). Pathology of peripheral artery disease in patients with critical limb ischemia. J. Am. Coll. Cardiol. 72, 2152–2163. doi: 10.1016/j.jacc.2018.08.002, PMID: 30166084

[B31] Oshima T. Ozono R. Yano Y. Oishi Y. Teragawa H. Higashi Y. . (2005). Association of Helicobacter pylori infection with systemic inflammation and endothelial dysfunction in healthy male subjects. J. Am. Coll. Cardiol. 45, 1219–1222. doi: 10.1016/j.jacc.2005.01.019, PMID: 15837252

[B32] Pasceri V. Patti G. Cammarota G. Pristipino C. Richichi G. Di Sciascio G. (2006). Virulent strains of Helicobacter pylori and vascular diseases: a meta-analysis. Am. Heart J. 151, 1215–1222. doi: 10.1016/j.ahj.2005.06.041, PMID: 16781222

[B33] Peluso I. Morabito G. Urban L. Ioannone F. Serafini M. (2012). Oxidative stress in atherosclerosis development: the central role of LDL and oxidative burst. Endocr. Metab. Immune Disord. Drug Targets. 12, 351–360. doi: 10.2174/187153012803832602, PMID: 23061409

[B34] Riad M. (2021). Association of Helicobacter pylori infection with coronary artery disease: is it an independent risk factor? Egypt Heart J. 73, 61. doi: 10.1186/s43044-021-00185-2, PMID: 34216301 PMC8254686

[B35] Rosenfeld M. E. Campbell L. A. (2011). Pathogens and atherosclerosis: update on the potential contribution of multiple infectious organisms to the pathogenesis of atherosclerosis. Thromb. Haemost. 106, 858–867. doi: 10.1160/TH11-06-0392, PMID: 22012133

[B36] Sawayama Y. Hamada M. Otaguro S. Maeda S. Ohnishi H. Fujimoto Y. . (2008). Chronic Helicobacter pylori infection is associated with peripheral arterial disease. J. Infect. Chemother. 14, 250–254. doi: 10.1007/s10156-008-0613-4, PMID: 18574664

[B37] Shi H. Li Y. Dong C. Si G. Xu Y. Peng M. . (2022). Helicobacter pylori infection and the progression of atherosclerosis: A systematic review and meta-analysis. Helicobacter. 27, e12865. doi: 10.1111/hel.12865, PMID: 34841620

[B38] Singh R. Lanchantin J. Sekhon A. Qi Y. (2017). Attend and predict: understanding gene regulation by selective attention on chromatin. Adv. Neural Inf Process Syst. 30, 6785–6795., PMID: 30147283 PMC6105294

[B39] Song P. Rudan D. Wang M. Chang X. Rudan I. (2019a). National and subnational estimation of the prevalence of peripheral artery disease (PAD) in China: a systematic review and meta-analysis. J. Glob Health 9, 010601. doi: 10.7189/jogh.09.010601, PMID: 30873278 PMC6377796

[B40] Song P. Rudan D. Zhu Y. Fowkes F. J. I. Rahimi K. Fowkes F. G. R. . (2019b). Global, regional, and national prevalence and risk factors for peripheral artery disease in 2015: an updated systematic review and analysis. Lancet Glob Health 7, e1020–e1e30. doi: 10.1016/S2214-109X(19)30255-4, PMID: 31303293

[B41] Sun L. Zheng H. Qiu M. Hao S. Liu X. Zhu X. . (2023). Helicobacter pylori infection and risk of cardiovascular disease. Helicobacter. 28, e12967. doi: 10.1111/hel.12967, PMID: 36974892

[B42] Temesgen G. B. Menon M. Gizaw S. T. Yimenu B. W. Agidew M. M. (2022). Evaluation of lipid profile and inflammatory marker in patients with gastric Helicobacter pylori infection, Ethiopia. Int. J. Gen. Med. 12, 271–278. doi: 10.2147/IJGM.S345649, PMID: 35023964 PMC8747762

[B43] Tsao C. W. Aday A. W. Almarzooq Z. I. Alonso A. Beaton A. Z. Bittencourt M. S. . (2022). Heart disease and stroke statistics-2022 update: A report from the american heart association. Circulation. 145, e153–e639. doi: 10.1161/CIR.0000000000001052, PMID: 35078371

[B44] Wang B. Yu M. Zhang R. Chen S. Xi Y. Duan G. (2020). A meta-analysis of the association between Helicobacter pylori infection and risk of atherosclerotic cardiovascular disease. Helicobacter 25, e12761. doi: 10.1111/hel.12761, PMID: 33026704

[B45] Xu Z. Li J. Wang H. Xu G. (2017). Helicobacter pylori infection and atherosclerosis: is there a causal relationship? Eur. J. Clin. Microbiol. Infect. Dis. 36, 2293–2301. doi: 10.1007/s10096-017-3054-0, PMID: 28752210

[B46] Ye J. Feng T. Su L. Li J. Gong Y. Ma X. (2023). Interactions between Helicobacter pylori infection and host metabolic homeostasis: A comprehensive review. Helicobacter. 28, e13030. doi: 10.1111/hel.13030, PMID: 37871913

[B47] Zheng W. Zhu S. Wang X. Chen C. Zhen Z. Xu Y. . (2025). Machine learning for early diagnosis of Kawasaki disease in acute febrile children: retrospective cross-sectional study in China. Sci. Rep. 15, 6799. doi: 10.1038/s41598-025-90919-y, PMID: 40000757 PMC11862119

[B48] Zhu S. Tan X. Huang H. Zhou Y. Liu Y. (2024). Data-driven rapid detection of Helicobacter pylori infection through machine learning with limited laboratory parameters in Chinese primary clinics. Heliyon. 10, e35586. doi: 10.1016/j.heliyon.2024.e35586, PMID: 39170567 PMC11336724

