# Supplementary material for: Interpretable machine learning analysis for relationships between Helicobacter pylori infection and peripheral atherosclerosis: a retrospective cohort study
Source: Front Cell Infect Microbiol. 2026 Feb 11;16:1693262. doi: 10.3389/fcimb.2026.1693262 (PMC12932622; doi:10.3389/fcimb.2026.1693262)

**Supplementary Table. 1 The Clinical characteristics of subgroups with/without H. pylori infections**

| **Variables** | **H. pylori-negative (n=3,351)** | **H. pylori-positive (n=2,511)** | ***P*-value** |
| --- | --- | --- | --- |
| Age (n, %) |  |  | **0.004**** |
| ≥60 | 472 (14.09%) | 424 (16.89%) |  |
| <60 | 2879 (85.91%) | 2087 (83.11%) |  |
| Gender (n, %) |  |  | 0.068 |
| Male | 2247 (67.05%) | 1741 (69.33%) |  |
| Female | 1104 (32.95%) | 770 (30.67%) |  |
| Smoke (n, %) |  |  | **0.022*** |
| Yes | 1003 (29.93%) | 823 (32.78%) |  |
| No | 2348 (70.07%) | 1688 (67.22%) |  |
| Drink (n, %) |  |  | 0.477 |
| Yes | 901 (26.89%) | 697 (27.76%) |  |
| No | 2450 (73.11%) | 1814 (72.24%) |  |
| White blood cell (10^9/L) | 5.55±0.64 | 5.56±0.65 | 0.640 |
| Haemoglobin (g/L) | 132.27±2.54 | 132.36±2.61 | 0.152 |
| Platelet (10^9/L) | 167.64±19.30 | 167.55±19.14 | 0.857 |
| C-reactive protein (mg/L) | 10.61±2.04 | 10.58±1.97 | 0.636 |
| Triglycerides (mmol/L) | 1.96±1.66 | 2.01±1.72 | 0.300 |
| Total cholesterol (mmol/L) | 5.07±0.95 | 5.06±0.94 | 0.654 |
| High density lipoprotein (mmol/L) | 1.39±0.30 | 1.38±0.28 | 0.079 |
| Low density lipoprotein (mmol/L) | 2.71±0.71 | 2.69±0.69 | 0.229 |
| Systolic blood pressure (mm/Hg) | 127.94±17.39 | 128.36±17.62 | 0.362 |
| Diastolic blood pressure (mm/Hg) | 77.33±11.66 | 77.44±11.53 | 0.707 |
| Fasting blood glucose (mmol/L) | 5.43±1.47 | 5.49±1.54 | 0.153 |
| Glycosylated hemoglobin A1c (%) | 5.88±0.89 | 5.93±0.94 | **0.034*** |
| Serum ferritin (μg/L) | 607.08±310.15 | 612.57±303.70 | 0.499 |
| Peripheral atherosclerosis (n, %) |  |  | **<0.001***** |
| Yes | 573 (17.10%) | 782 (31.14%) |  |
| No | 2778 (82.90%) | 1729 (68.86%) |  |

Note: *, p.value <0.05, **, p.value <0.01, ***, p.value <0.001. Helicobacter pylori, H. pylori.

**Supplementary Table 2. Univariate logistic regression analysis of risk factors for peripheral atherosclerosis.**

| **Variable** | **OR** | **OR_CI_Lower** | **OR_CI_Upper** | **p_value** |
| --- | --- | --- | --- | --- |
| **H. pylori*** | **2.19** | **1.94** | **2.48** | **1.04E-35** |
| **Sex*** | **3.82** | **3.24** | **4.52** | **6.71E-56** |
| **Age*** | **23.70** | **19.86** | **28.40** | **4.89E-264** |
| **Smoke*** | **2.94** | **2.59** | **3.34** | **2.04E-63** |
| **Drink*** | **9.31** | **8.13** | **10.68** | **2.95E-225** |
| WBC | 1.04 | 0.95 | 1.14 | 0.379 |
| HB | 1.01 | 0.99 | 1.03 | 0.408 |
| PLT | 1.00 | 0.99 | 1.00 | 0.709 |
| **CRP*** | **1.09** | **1.06** | **1.13** | **3.55E-09** |
| TG | 1.03 | 0.99 | 1.06 | 0.098 |
| **TC*** | **1.25** | **1.17** | **1.33** | **6.58E-12** |
| HDL | 1.13 | 0.92 | 1.39 | 0.233 |
| **LDL*** | **1.34** | **1.23** | **1.46** | **2.73E-11** |
| **HbA1c*** | **1.33** | **1.26** | **1.42** | **6.41E-21** |
| **FBG*** | **1.17** | **1.13** | **1.22** | **1.36E-17** |
| **DBP*** | **1.02** | **1.02** | **1.03** | **1.78E-19** |
| **SBP*** | **1.03** | **1.03** | **1.03** | **2.82E-58** |
| SF | 1.00 | 1.00 | 1.00 | 0.145 |

**Note: w**hite blood cell (WBC), haemoglobin (HB), platelet (PLT), C-reactive protein (CRP), fasting blood glucose (FBG), glycated hemoglobin A1c (HbA1c), triglyceride (TG), total cholesterol (TC), low-density lipoprotein (LDL), and high-density lipoprotein (HDL), serum ferritin (SF), systolic blood pressure (SBP), and diastolic blood pressure (DBP). *, p_value <0.05.

**Supplementary Table 3. Multivariate logistic regression analysis of risk factors for peripheral atherosclerosis.**

| **Variable** | **OR** | **OR_CI_Lower** | **OR_CI_Upper** | **p_value** |
| --- | --- | --- | --- | --- |
| **H. pylori (+)*** | **5.27** | **4.27** | **6.54** | **1.73E-52** |
| **Male*** | **5.88** | **4.22** | **8.29** | **7.28E-25** |
| Age >60 | 4.85E-12 | 8.99E-106 | 3.68E-97 | 0.931 |
| **Smoking*** | **2.11** | **1.67** | **2.67** | **3.48E-10** |
| Drink (+) | 5.68E+8 | 6.37E+92 | 4.55E+100 | 0.997 |
| CRP | 1.00 | 0.99 | 1.01 | 0.973 |
| TC | 1.04 | 0.85 | 1.27 | 0.701 |
| **LDL*** | **2.10** | **1.47** | **3.04** | **5.89E-05** |
| HbA1c | 1.03 | 0.84 | 1.26 | 0.794 |
| FBG | 1.00 | 0.88 | 1.13 | 0.996 |
| DBP | 1.01 | 1.00 | 1.03 | 0.088 |
| SBP | 1.00 | 0.99 | 1.01 | 0.477 |

**Note:** C-reactive protein (CRP), fasting blood glucose (FBG), glycated hemoglobin A1c (HbA1c), triglyceride (TG), total cholesterol (TC), low-density lipoprotein (LDL), and systolic blood pressure (SBP). *, p_value <0.05.

**Supplementary Table 4. Comparison of Clinical characteristics among different Peripheral atherosclerosis prognosis subgroups.**

| **Variables** | **Persistent Negative (n=4,074)** | **Emerging PA (n=433)** | **Persistent PA (n=1,355)** | ***P*-value** |
| --- | --- | --- | --- | --- |
| **Age** **(n, %)** |  |  |  | **0.005**** |
| ≥60 | 583 (14.31%) | 70 (16.17%) | 243 (17.93%) |  |
| <60 | 3491 (85.69%) | 363 (83.83%) | 1112 (82.07%) |  |
| **Gender (n, %)** |  |  |  | **<0.001***** |
| Male | 2710 (66.52%) | 323 (74.60%) | 955 (70.48%) |  |
| Female | 1337 (33.48%) | 110 (25.40%) | 400 (29.52%) |  |
| **Smoke (n, %)** |  |  |  | **<0.001***** |
| Yes | 1197 (29.38%) | 173 (39.95%) | 456 (33.65%) |  |
| No | 2877 (70.62%) | 260 (60.05%) | 899 (66.35%) |  |
| **Drink (n, %)** |  |  |  | **<0.001***** |
| Yes | 1037 (25.45%) | 132 (30.48%) | 429 (31.66%) |  |
| No | 3037 (74.55%) | 301 (69.52%) | 926 (68.34%) |  |
| **H. pylori infection (n, %)** |  |  |  | **0.035*** |
| Yes | 1700 (41.73%) | 197 (45.50%) | 614 (45.31%) |  |
| No | 2374 (58.27%) | 236 (54.50%) | 741 (54.69%) |  |
| **White blood cell (10^9/L)** | 5.50±0.48 | 6.16±1.36 | 5.54±0.62 | **<0.001***** |
| **Haemoglobin (g/L)** | 132.13±2.09 | 134.20±4.92 | 132.22±2.56 | **<0.001***** |
| **Platelet (10^9/L)** | 166.17±15.71 | 188.38±37.37 | 167.20±18.36 | **<0.001***** |
| **C-reactive protein (mg/L)** | 10.43±1.94 | 10.57±2.24 | 11.11±2.05 | **<0.001***** |
| **Triglycerides (mmol/L)** | 1.78±1.15 | 3.66±3.75 | 2.05±1.66 | **<0.001***** |
| **Total cholesterol (mmol/L)** | 5.00±0.91 | 5.36±1.18 | 5.15±0.96 | **<0.001***** |
| High density lipoprotein (mmol/L) | 1.39±0.29 | 1.29±0.31 | 1.40±0.30 | 0.194 |
| **Low density lipoprotein (mmol/L)** | 2.69±0.69 | 2.68±0.83 | 2.76±0.70 | **0.004**** |
| **Systolic blood pressure (mm/Hg)** | 127.76±17.43 | 130.01±17.77 | 128.58±17.54 | **0.021*** |
| **Diastolic blood pressure (mm/Hg)** | 77.07±11.56 | 78.97±11.72 | 77.78±11.64 | **0.002**** |
| **Fasting blood glucose (mmol/L)** | 5.41±1.42 | 5.64±1.72 | 5.54±1.64 | **0.001**** |
| **Glycosylated hemoglobin A1c (%)** | 5.87±0.88 | 6.02±1.06 | 5.95±0.97 | **<0.001***** |
| Serum ferritin (μg/L) | 607.61±306.81 | 634.5±319.41 | 606.89±305.09 | 0.211 |

Note:*, p.value <0.05;**, p.value <0.01; ***, p.value <0.001. peripheral atherosclerosis, PA; Helicobacter pylori, H. pylori.

**Supplementary Table 5. The distribution of model’s metrics from 14 Machine learning algorithms based on all clinical parameters.**

| **Models** | **Sensitivity** | **Specificity** | **Accuracy** | **PPV** | **NPV** | **F1 score** | **Youden's index** |
| --- | --- | --- | --- | --- | --- | --- | --- |
| Random Forest | 0.922 | 0.976 | 0.963 | 0.919 | 0.976 | 0.921 | 0.898 |
| Gradient Boosting | 0.918 | 0.975 | 0.962 | 0.918 | 0.975 | 0.918 | 0.893 |
| SVM Kernel | 0.892 | 0.97 | 0.951 | 0.898 | 0.967 | 0.895 | 0.861 |
| Logistic Model | 0.825 | 0.938 | 0.912 | 0.801 | 0.947 | 0.813 | 0.763 |
| NeighborMethod | 0.092 | 0.958 | 0.757 | 0.398 | 0.777 | 0.15 | 0.05 |
| PLS Model | 0.023 | 0.996 | 0.77 | 0.647 | 0.771 | 0.045 | 0.019 |
| BoostingMethod | 0.921 | 0.974 | 0.962 | 0.914 | 0.976 | 0.918 | 0.895 |
| Neural Network | 0.909 | 0.95 | 0.94 | 0.845 | 0.972 | 0.876 | 0.858 |
| Bayes Method | 0.806 | 0.891 | 0.872 | 0.692 | 0.938 | 0.744 | 0.697 |
| Discriminant Model | 0.87 | 0.942 | 0.925 | 0.82 | 0.96 | 0.844 | 0.812 |
| Lasso | 0.828 | 0.941 | 0.915 | 0.81 | 0.948 | 0.819 | 0.769 |
| Adaptive Boosting | 0.91 | 0.979 | 0.963 | 0.929 | 0.973 | 0.919 | 0.889 |
| **CATBoost** | **0.954** | **0.990** | **0.981** | **0.966** | **0.986** | **0.960** | **0.944** |
| LightGBM | 0.981 | 0.998 | 0.994 | 0.995 | 0.994 | 0.988 | 0.980 |

**Supplementary Table 6. The distribution of model’s metrics from 14 Machine learning algorithms based on critical parameters in Training sets.**

| **Models** | **Sensitivity** | **Specificity** | **Accuracy** | **PPV** | **NPV** | **F1 score** | **Youden's index** |
| --- | --- | --- | --- | --- | --- | --- | --- |
| Random Forest | 0.267 | 0.945 | 0.787 | 0.593 | 0.810 | 0.368 | 0.212 |
| Gradient Boosting | 0.270 | 0.948 | 0.791 | 0.612 | 0.811 | 0.375 | 0.218 |
| SVM Kernel | 0.227 | 0.956 | 0.787 | 0.610 | 0.804 | 0.331 | 0.183 |
| Logistic Model | 0.157 | 0.970 | 0.781 | 0.613 | 0.792 | 0.249 | 0.127 |
| NeighborMethod | 0.326 | 0.917 | 0.779 | 0.541 | 0.818 | 0.407 | 0.242 |
| PLS Model | 0.049 | 0.991 | 0.772 | 0.618 | 0.775 | 0.091 | 0.040 |
| BoostingMethod | 0.282 | 0.946 | 0.792 | 0.612 | 0.813 | 0.386 | 0.228 |
| Neural Network | 0.296 | 0.941 | 0.791 | 0.603 | 0.816 | 0.397 | 0.237 |
| Bayes Method | 0.374 | 0.898 | 0.777 | 0.527 | 0.826 | 0.437 | 0.272 |
| Discriminant Model | 0.197 | 0.962 | 0.785 | 0.612 | 0.799 | 0.299 | 0.160 |
| Lasso | 0.139 | 0.974 | 0.780 | 0.614 | 0.789 | 0.226 | 0.112 |
| Adaptive Boosting | 0.271 | 0.950 | 0.793 | 0.622 | 0.812 | 0.377 | 0.221 |
| **CATBoost** | **0.342** | **0.925** | **0.785** | **0.621** | **0.825** | **0.441** | **0.279** |
| LightGBM | 0.000 | 1.000 | 0.768 | NA | 0.768 | NA | 0.000 |

**Supplementary Table 7. The distribution of model’s metrics from 14 Machine learning algorithms based on critical parameters in Validation sets.**

| **Models** | **Sensitivity** | **Specificity** | **Accuracy** | **PPV** | **NPV** | **F1 score** | **Youden's index** |
| --- | --- | --- | --- | --- | --- | --- | --- |
| Random Forest | 0.270 | 0.943 | 0.789 | 0.586 | 0.813 | 0.370 | 0.214 |
| Gradient Boosting | 0.263 | 0.946 | 0.790 | 0.592 | 0.812 | 0.364 | 0.209 |
| SVM Kernel | 0.236 | 0.952 | 0.788 | 0.594 | 0.807 | 0.337 | 0.188 |
| Logistic Model | 0.161 | 0.964 | 0.780 | 0.570 | 0.795 | 0.251 | 0.125 |
| NeighborMethod | 0.325 | 0.916 | 0.781 | 0.535 | 0.820 | 0.404 | 0.241 |
| PLS Model | 0.055 | 0.990 | 0.776 | 0.629 | 0.779 | 0.100 | 0.045 |
| BoostingMethod | 0.278 | 0.940 | 0.789 | 0.580 | 0.814 | 0.376 | 0.218 |
| Neural Network | 0.280 | 0.934 | 0.784 | 0.557 | 0.814 | 0.373 | 0.214 |
| Bayes Method | 0.372 | 0.897 | 0.777 | 0.517 | 0.828 | 0.433 | 0.269 |
| Discriminant Model | 0.206 | 0.954 | 0.783 | 0.572 | 0.802 | 0.303 | 0.160 |
| Lasso | 0.154 | 0.965 | 0.779 | 0.564 | 0.793 | 0.242 | 0.118 |
| Adaptive Boosting | 0.261 | 0.944 | 0.787 | 0.580 | 0.811 | 0.360 | 0.204 |
| **CATBoost** | **0.313** | **0.925** | **0.785** | **0.553** | **0.819** | **0.399** | **0.237** |
| LightGBM | 0.000 | 1.000 | 0.771 | NA | 0.771 | NA | 0.000 |


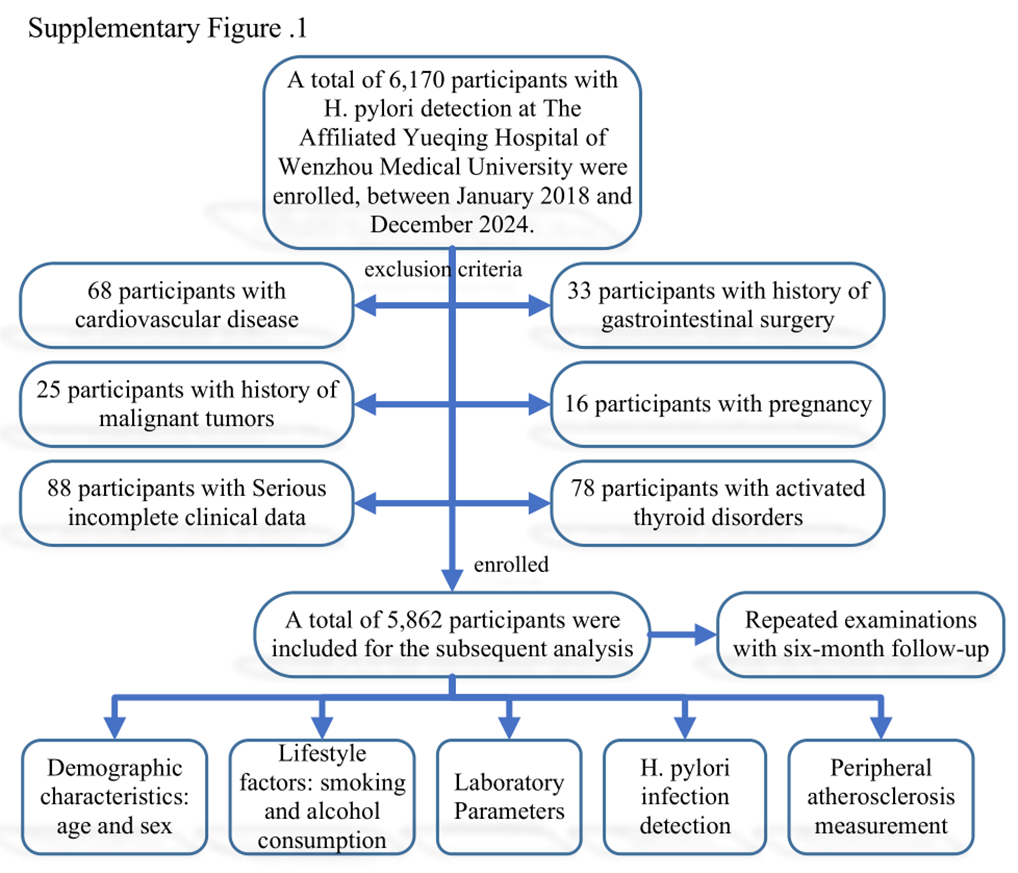


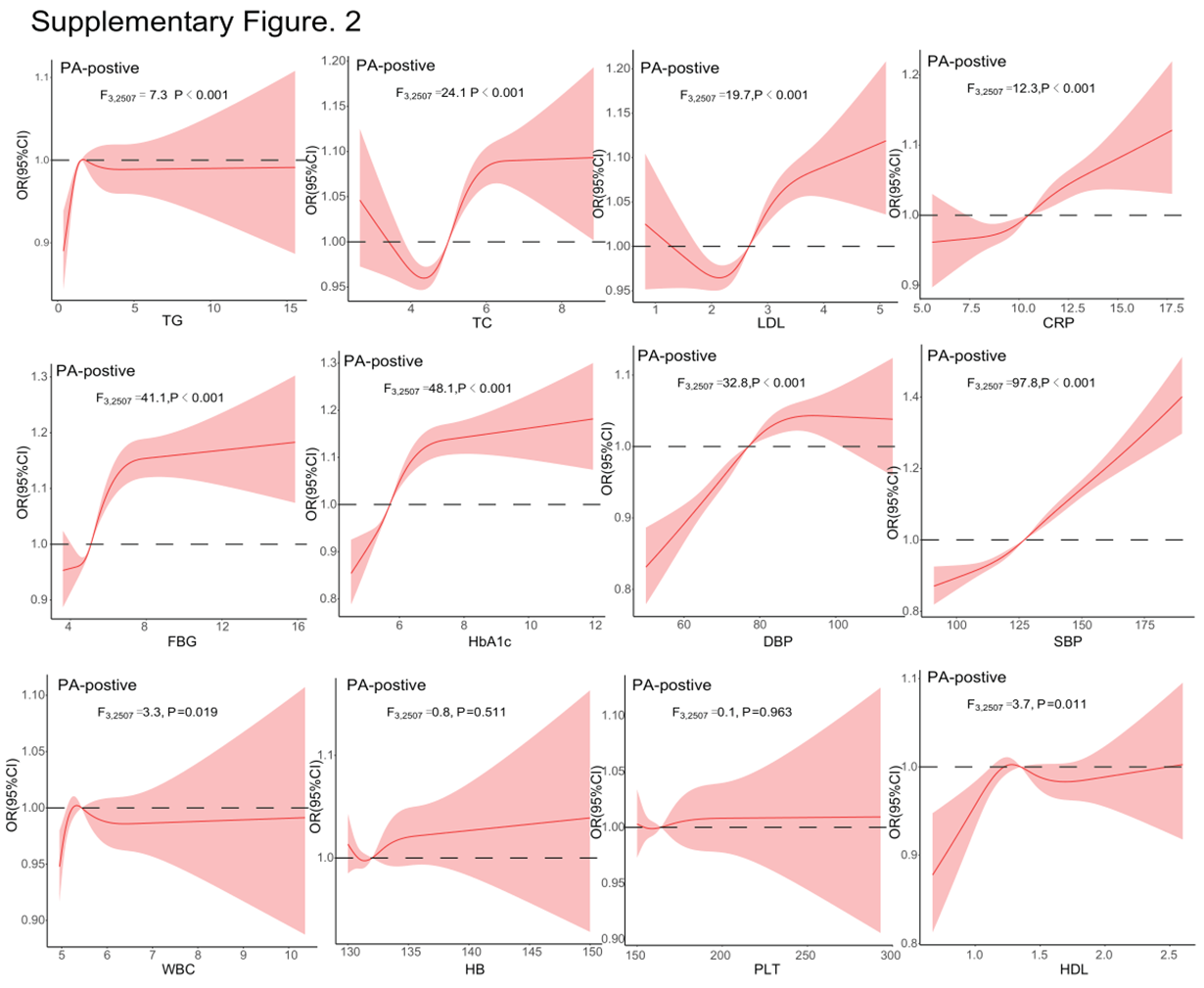


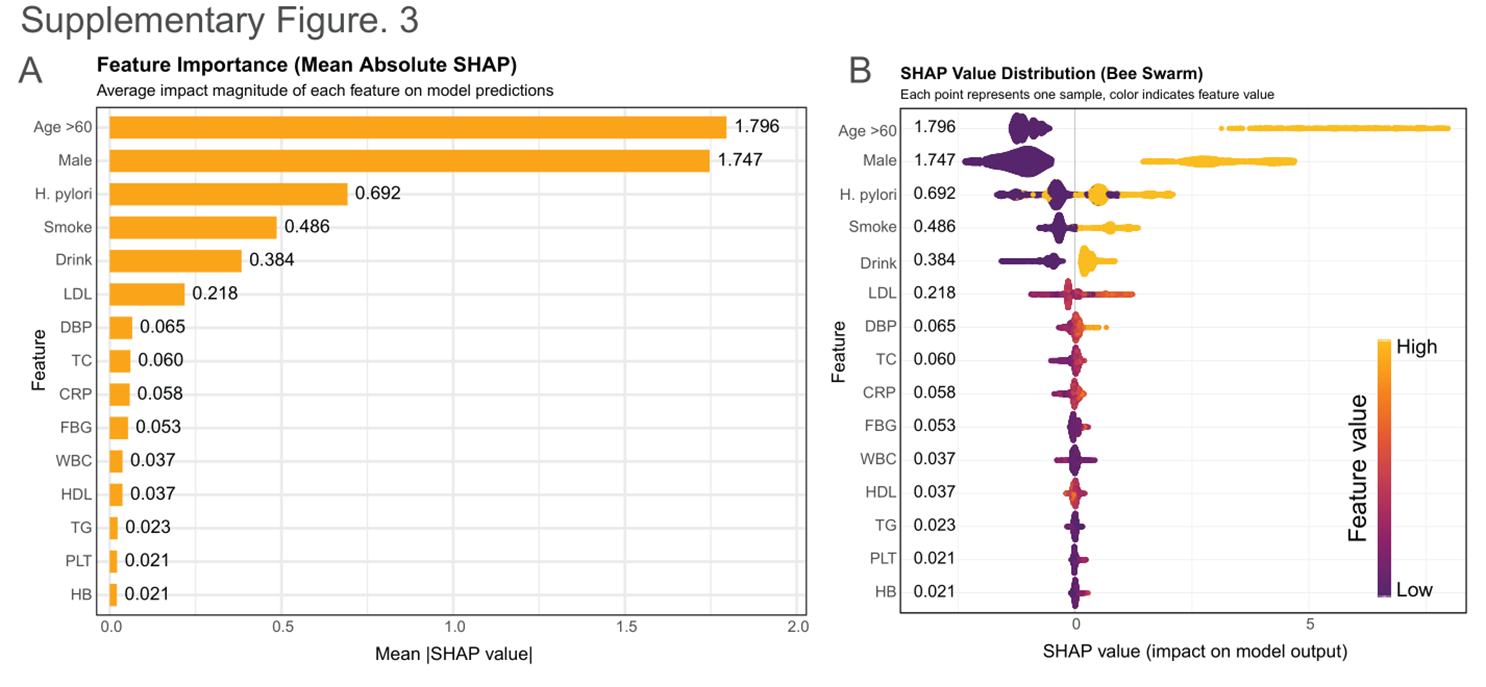

Supplement: Supplementary file 1 [file Table1.docx]
